# Ginseng of different ages is affected by the accumulation of heavy metals in ginseng soil

**DOI:** 10.1371/journal.pone.0269238

**Published:** 2022-06-13

**Authors:** Juxin Yin, Jianjian Zhuang, Xin Zhang, Chaojian Xu, Shaowu Lv

**Affiliations:** 1 School of Information and Electrical Engineering, Zhejiang University City College, Hangzhou, People’s Republic of China; 2 Department of Clinical Pharmacology, Key Laboratory of Clinical Cancer Pharmacology and Toxicology Research of Zhejiang Province, Affiliated Hangzhou First People’s Hospital, Cancer Center, Zhejiang University School of Medicine, Hangzhou, Zhejiang, China; 3 Key Laboratory for Molecular Enzymology and Engineering of the Ministry of Education, College of Life Science, Jilin University, Changchun, China; Universidade de Coimbra, PORTUGAL

## Abstract

Heavy-metal pollution has been established to affect ginseng quality. However, this effect is still unknown in ginseng of different ages, emphasizing the need to investigate the effects of heavy metals in soils on ginseng growth. Herein, we determined the content of heavy metals (Cu, Cd, Pb, Hg, and As) in ginseng of different ages (2 to 6-year-old) and the corresponding soil samples. Then, the total ginsenosides content of ginseng and rate-limiting enzyme (HMGR, SQE, CYP450) activity in the synthesis of ginsenosides were assessed. Results from 200 differently-aged Chinese ginseng showed that increased ginsenoside content in 3 to 5-year-old ginseng was paralleled by increased heavy metal element content in ginseng and its soil. The activity of rate-limiting enzymes increased in the first four years of ginseng growth and then exhibited a steady or downward trend. Further analysis suggested that heavy metal elements in soils could directly affect ginsenoside content. Moreover, we found that Cu significantly affected the rate-limiting enzyme CYP450 activity. Further principal component analysis and correlation analysis found that heavy metals could obviously inhibit ginseng growth during the 5th and 6th years. Heavy metal content in soils has huge prospects for predicting ginsenoside, Cu and As content in ginseng. This study provided support for ginseng cultivation, quality research and quality assessment.

## Introduction

Panax ginseng C. A. Meyer (Asian ginseng) is a traditional Chinese herbal medicine with nutritional and medicinal value, rich in ginsenosides, polysaccharides and other active components, and widely used by people all over the world [1, 2]. Over the last decade, ginseng and its products have been extensively used for disease treatment and improving human health [3, 4]. In this regard, with the increase in demand in the pharmaceutical and health markets, much emphasis has been placed on ensuring its safety for consumers [5].

It has been established that heavy metal contamination from lead, mercury, arsenic (As) or cadmium is one of the main abiotic stresses leading to plant health defects and can seriously affect plant product safety [6, 7]. At present, it is strongly recommended by the World Health Organization to check for the presence of heavy metals in Chinese medicine (from the raw materials to the final products), given that increasing evidence corroborates that heavy metals can disrupt normal body functions and produce serious health hazards [8].

The main source of heavy metal pollution is soil residues. In this regard, the application of pesticides containing heavy metals and mining activities can directly affect the safe production and development of traditional Chinese medicine [9]. Given their high reactivity, heavy metals can lead to inhibition of root growth and resulted in an increase in protein oxidation [10]. Heavy metals can induce oxidative stress directly and affect plant growth, senescence and energy synthesis through ROS oxidative stress pathway [11] and jasmonic acid pathway [12]. It is widely acknowledged that ginseng grows in humus-rich soil with pH 5.0 ~ 6.0, and heavy metals are dominant in this kind of acidic environment. Importantly, some heavy metals have been detected in root extracts or commercial preparations. For example, a study by Rubio et al. [13] found major toxic metals Al, Pb and Cd in 20 ginseng samples ginseng in Europe. Similarly, Filipiak-Szok et al. [14] detected heavy metals Pb, Cd, As, Ba and Sb in raw ginseng plant material and dietary supplements in Asian and European markets.

Heavy metals such as Cd and Pb are well-recognized to be present in soil environments in high concentrations and can affect plant nutrient composition [15]. In this respect, heavy metals can reportedly alter ginseng’s biological activity and affect ginsenoside synthesis by regulating enzyme activity during ginseng growth [16, 17]. Ali et al. found that various ginseng antioxidant enzymes were inhibited under different Cu concentrations (10 μM~50 μM), substantiating that ginseng can grow under copper stress by regulating enzyme activity [18]. Zhang et al. reported that Fe was toxic to Panax ginseng and can cause Rusty-Root Symptom [19]. Interestingly, Ziarati et al. compared heavy metal contents from markets in Tehran and Beijing and found that heavy metals could contaminate ginseng at different transportation and storage stages [20]. Durgnat et al found that Hg, and As were present in most Ginseng extracts samples [6]. Overall, these findings demonstrate that heavy metals can directly influence the quality of ginseng.

To ensure the safety and wide application of ginseng, a better understanding of the role of heavy metals in ginseng and its growing environment, especially for heavy metals in ginseng raw material, is needed. To the best of our knowledge, few studies have documented the accumulation of heavy metals in ginseng of different ages and its growth soils, nor is it clear whether heavy metals can affect the activity of key enzymes for ginsenoside synthesis. Therefore, in this study, we used 5 common heavy metals(Cu, Cd, Pb, Hg, and As), which is required to be measured by the Joint FAO/WHO Department of Essential Drugs and Medicines Policy (1999) and investigated changes in the concentration of these heavy metals in ginseng of different ages and the growth soils. We also assessed the activity of ginsenosides and their rate-limiting enzymes and analyzed the correlation between these factors. The findings of the present study refine our knowledge on conditions that affect ginseng growth and may be used for its safe cultivation.

## Materials and methods

### Materials

All standard substances were obtained from the National Institute of Metrology, China, while other materials were obtained from Sangon Biotech (Shanghai, China). Diethyldithiocarbamate trihydrate (cas:20624-25-3,(C_2_H_5_)_2_NCSSNa·3H2O, DDTC) and 4-methyl-2-pentanone(cas:108-10-1, (CH_3_)_2_CHCH_2_COCH_3_) were obtained from Aladdin (Shanghai, China) and Sodium borohydride(cas:16940-66-2, NaBH_4_) was purchased from Sigma. 200 differently-aged Chinese ginseng (*Panax ginseng C*.*A*. *Meyer*) (2–6 years) and surface soil (0–0.15 m) naturally co-contaminated with Cu, Pb, Cd, As, and Hg were collected in June 2017 from a planted area in the northeast of Jilin City, Jilin Province, China (latitude is N, 43.8°; longitude is E, 126.5°).

### Sample preparation

Samples were prepared by wet digestion. All glassware and PTFE digestion inner tanks were soaked in nitric acid overnight and repeatedly rinsed with distilled water. Ginseng roots were cleaned with ultrapure water and cut into thin slices. After oven drying at 80°C, the slices were ground into powder and passed through a 2 mm mesh sieve for further analysis. For the ginseng soils, root debris was carefully removed, and the soil passed through a 2 mm mesh sieve for further analysis. 0.5 g powder samples were placed into 3 beakers, 2 mL of nitric acid was added to each beaker with a pipette and dissolved by heating. Upon dissolution, 4 mL of nitric acid and 2 mL of hydrochloric acid were added. The heating continued until white smoke production stopped, indicating that digestion was complete (the solution had become clear and transparent). Samples were made up to a constant volume with ultrapure water.

### Heavy metals determination

A Cu standard series solution was introduced into a Flame atomic absorption spectrophotometer according to the order of mass concentration from low to high, and the standard curve was prepared. Cu in ginseng soil and roots were analyzed under the same conditions, i.e., wavelength 324.8 nm, slit 0.7 nm, lamp current 6 mA, flame type C_2_H_2_-air. Pb and Cd were measured using a graphite furnace atomic absorption spectrophotometry. The Pb wavelength was 283.3 nm, slit 0.7 nm, lamp current 5 mA, flame type C_2_H_2_-air. The Cd wavelength was 228.8 nm, slit 0.7 nm, lamp current 5 mA, flame type C_2_H_2_-air. As and Hg were measured using an AFS-230E atomic fluorescence spectrophotometer (Haiguang Instrument, Beijing, China). For As measurement, the conditions were: voltage: 300V, lamp current 30mA, hollow cathode lamp, sample volume 1.5mL, carrier gas flow rate 400mL/min. For Hg measurement, the conditions were: voltage 280V, lamp current 30mA, hollow cathode lamp, sample volume 1.5ml, carrier gas flow rate 400mL/min.

### Total ginsenosides determination

10 mL of 70% ethanol was added to a 1 g ginseng root sample and shaken at 100 r/min overnight. Then ultrasonication was performed at 40 kHz for 30 min, followed by rotary evaporation. Ten mL of distilled water and 10 mL of diethyl ether were added to the evaporated flask. The solution was poured into a separatory funnel to degrease, and the aqueous layer was taken. The extraction was repeated three times by adding 10 mL of water-saturated n-butanol. The sugar was washed by adding 10 mL of distilled water to obtain an organic layer solution. After rotary evaporation, the solution was dissolved in 10 mL of chromatographically pure methanol and stored in a brown bottle.

For sample determination, 100 μL was added to the tube and placed in a 65° C water bath until the solution was completely evaporated. The test tube was removed from the water bath, 0.2 ml of the prepared 5% vanillin-glacial acetic acid solution was added to the fume hood, then 0.8 mL of perchloric acid was added, and the tube was heated in a constant temperature water bath at 60°C. After 15 min, it was quickly cooled with ice water, 5 mL of glacial acetic acid was added, and the mixture was shaken. The absorbance at 560 nm of the solution in the test tube was then measured.

### Enzyme activity assay

The activity of three enzymes was determined using different methods. For HMGR, the activity was measured using an HMG-CoA Reductase Assay Kit (Sigma-Aldrich). The activity of SE was measured with a Plant Squalene epoxidase ELISA Kit (TSZ, Framingham, MA, USA). The P450 activity was determined by the NADPH subtraction method. The reaction mixture was a total volume of 1 mL and contained 0.3 mM tetradecanoic acid solution in DMSO (final concentration, 2%) and 20 μg crude enzyme in 50 mM potassium-phosphate buffer (pH 7.5). The reaction was initiated upon the addition of 100 μL of a 1.5 mM aqueous NADPH solution, followed at 340 nm ("NADPH = 6.22 mM^-1^cm^-1^), and lasted 5 min. The enzyme activity was uniformly standardized to U/ ginseng.

### Correlation analysis and PCA

The analysis results of heavy metals in this study were presented as means (c). Correlations were analyzed among heavy metal elements in soils and ginseng of different ages. A PCA model was used to predict changes in various ginseng elements, including ginsenoside content, heavy metal content, and activity of rate-limiting enzymes. Correlation analysis and PCA were conducted using MATLAB ^®^.

## Results

### Temporal variation in total ginsenosides, heavy metal contents and enzyme activity

**Fig 1:**
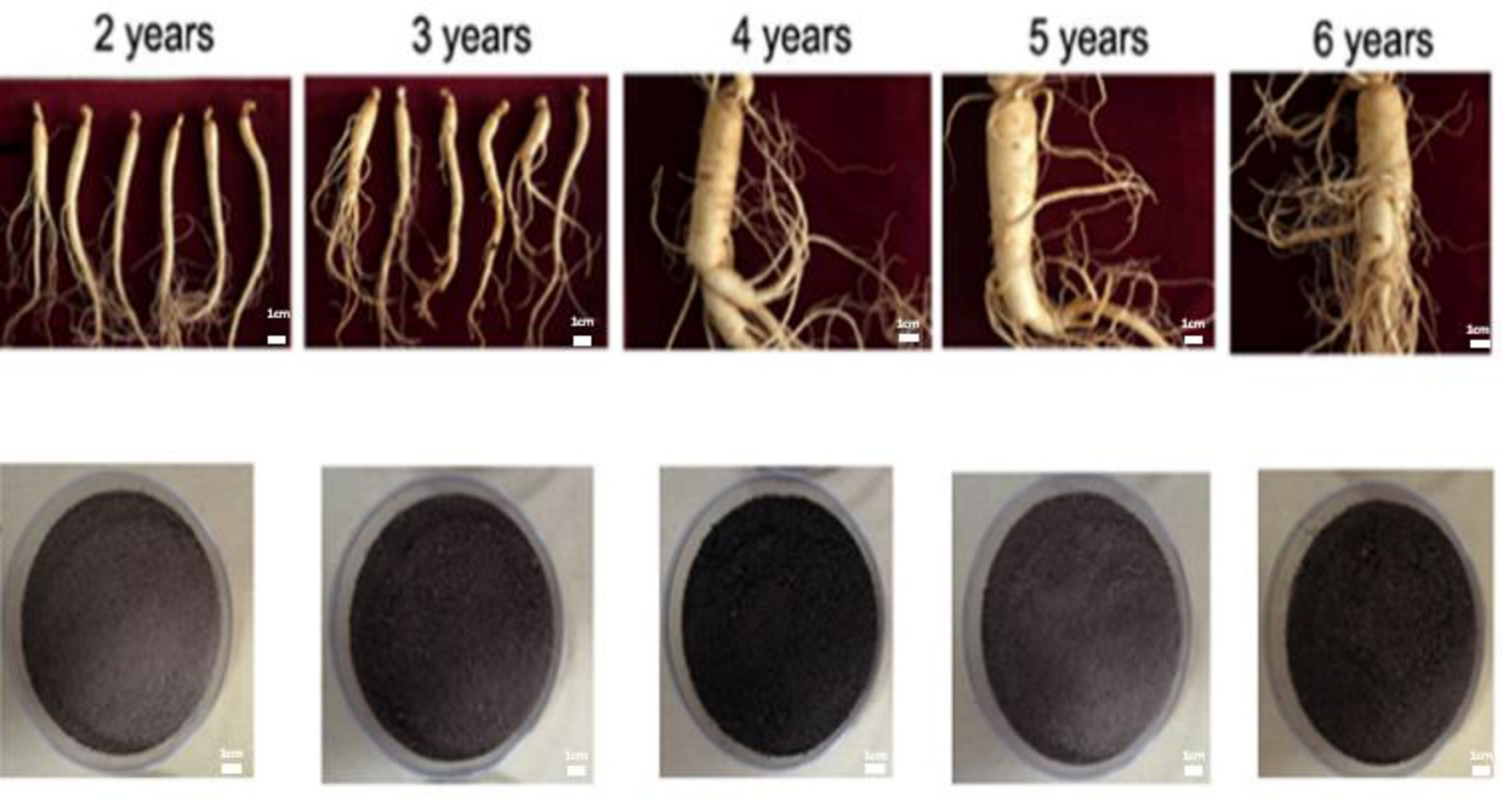
Representative ginseng and its associated soil over time. Ginseng and its associated soil in 2–6 years collected from some large ginseng farm in Jilin Province, northeast of China.

**Fig 2:**
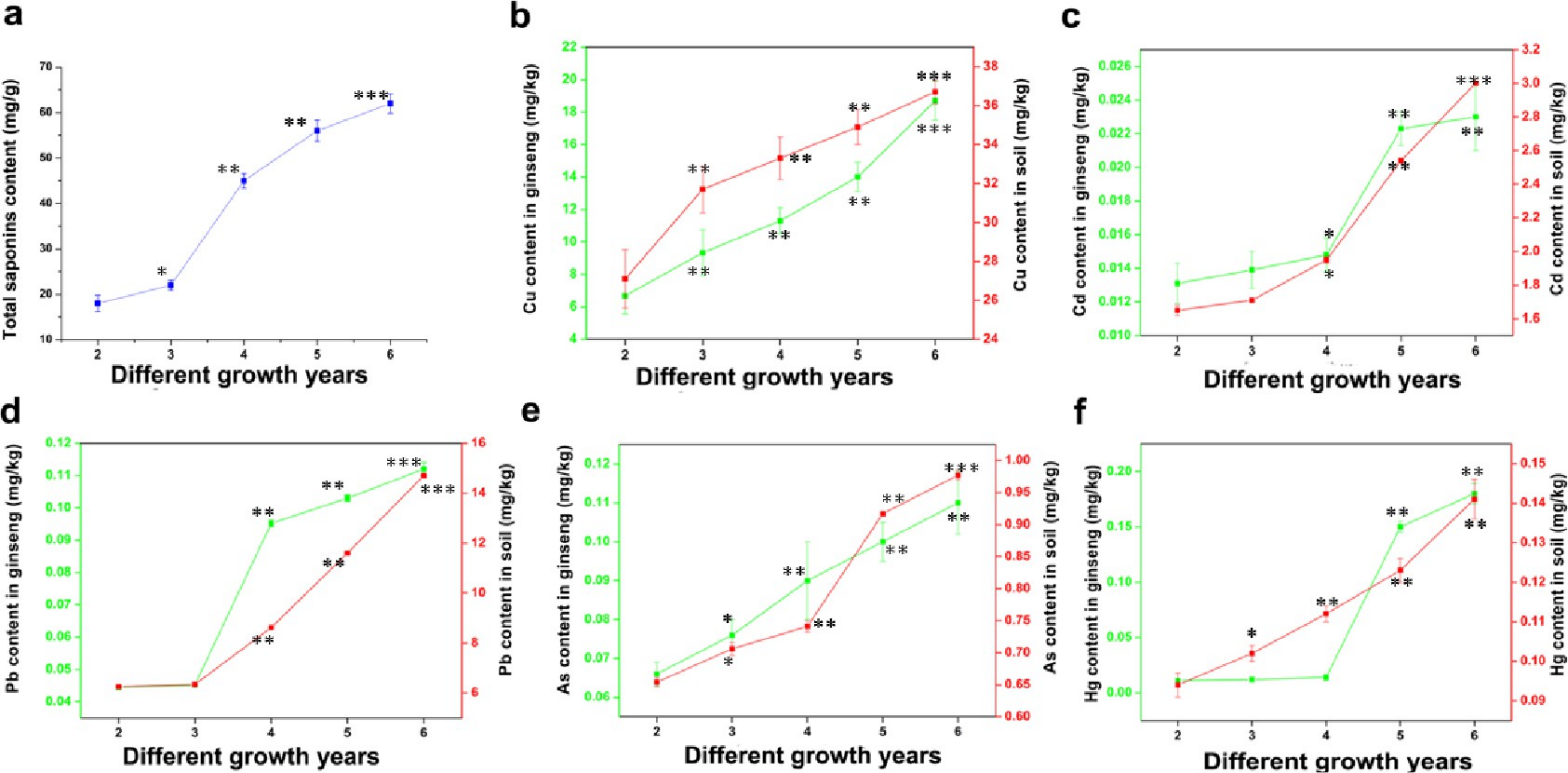
Contents of total ginsenosides and five heavy metal elements (Cu, Cd, Hg, As, Pb) in annual samples. The green Y axis on the left represents the content of heavy metal elements in ginseng, and the red Y axis on the right represents the content of heavy metal elements in ginseng soil. a: Changes in the content of total ginsenosides of ginseng in 2–6 years. b~f: changes of heavy metals Cu, Cd, Pb, As, Hg in Ginseng and its growing soil in 2–6 years; *p<0.05 vs 2 years, **p<0.01 vs 2 years, ***p<0.001 vs 2 years.

Northeast China is well-established as one of the important global ginseng production areas. In recent years, excessive heavy metal pollution in this region has caused a serious supply and demand imbalance in ginseng export. Therefore, ginseng and its associated soils in Northeast China (Jilin province) were selected for our research (Fig 1). In the present study, we investigated changes in total ginsenosides in ginseng of different ages. We found that the total ginsenosides content accumulated every year and exhibited the fastest increase from 3 to 5 years (Fig 2A), consistent with changes in ginseng appearance (Fig 1), suggesting that this is the period where ginseng grows the most vigorously.

Heavy metal stress is one of the main abiotic stresses leading to plant health defects [21, 22]. In our study, we first established standard curves for five heavy metal elements (S1 Fig), and all detection methods yielded a good linear relationship. The limit of detection for the five elements were: Cu 0.5, Pb 0.04, Cd 0.03, As 0.01, and Hg 0.02 mg/kg. The changes in the five heavy metals Cu, Cd, Pb, As, and Hg contents in ginseng of different ages and its growing soil are illustrated in Fig 2B–2F, respectively. We found that ginseng absorbed the five heavy metal elements from the soil through their roots during the 2–6 years of ginseng growth. Moreover, the content of the five heavy metals, both in the soil and in ginseng, increased annually, suggesting a gradual accumulation of heavy metals in the soil. Among the five heavy metals, Cu content was the highest in ginseng and its soil, most likely from human factors such as fertilizer spraying. Interestingly, compared to the limits established by the Joint FAO/WHO Department of Essential Drugs and Medicines Policy (1999) (Cu<20 mg/kg, As <3 mg/kg, Pb <10 mg/kg, Cd <0.3 mg/kg, Hg <0.1 mg/kg) [6], levels of all heavy metals detected in ginseng were within the standard requirements.

**Fig 3:**
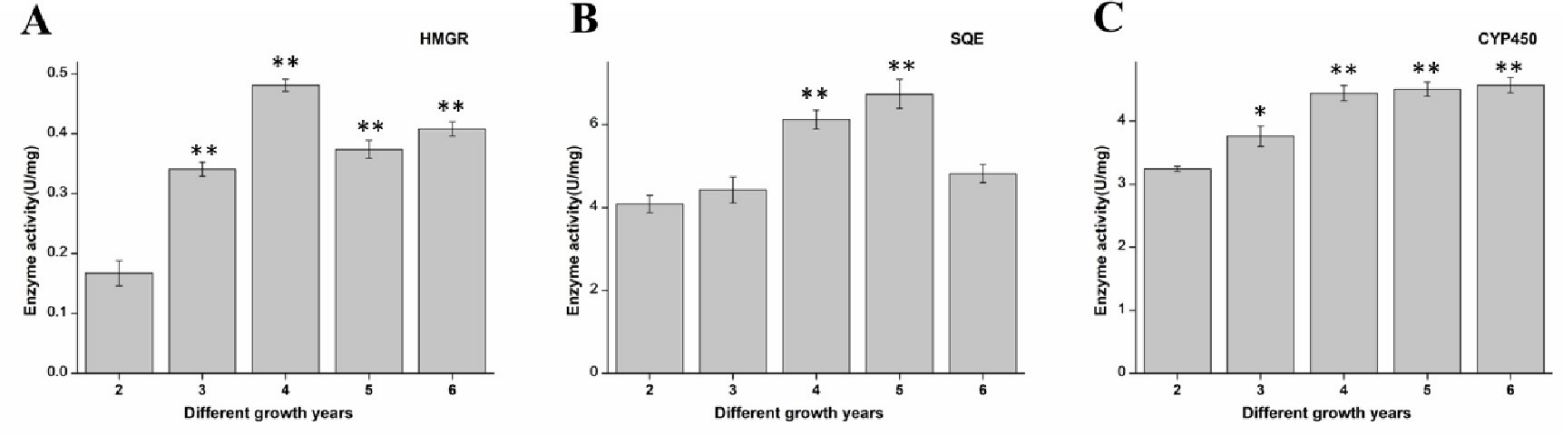
Enzyme activity in ginseng in different years. A: HMGR enzyme activity. B: SQE enzyme activity. C: CYP450 enzyme activity. The enzyme activity was uniformly standardized to U/ ginseng; *p<0.05 vs 2 years, **p<0.01 vs 2 years.

There is overwhelming evidence that heavy metals can also affect ginsenoside synthesis by affecting enzyme activity [23, 24]. Accordingly, we measured the activity of these rate-limiting enzymes. As shown in Fig 3, the activity of HMGR enzymes was relatively high in 4-year-old ginseng, and the activity of SQE enzymes was relatively high in 5-year-old ginseng. Moreover, the activity of CYP450 enzymes increased during the 3rd to 4th year and then remained at a stable level. Therefore, changes in their activities were not consistent, suggesting that the impact of heavy metals on their activities was different.

### Correlation analysis and PCA

**Fig 4:**
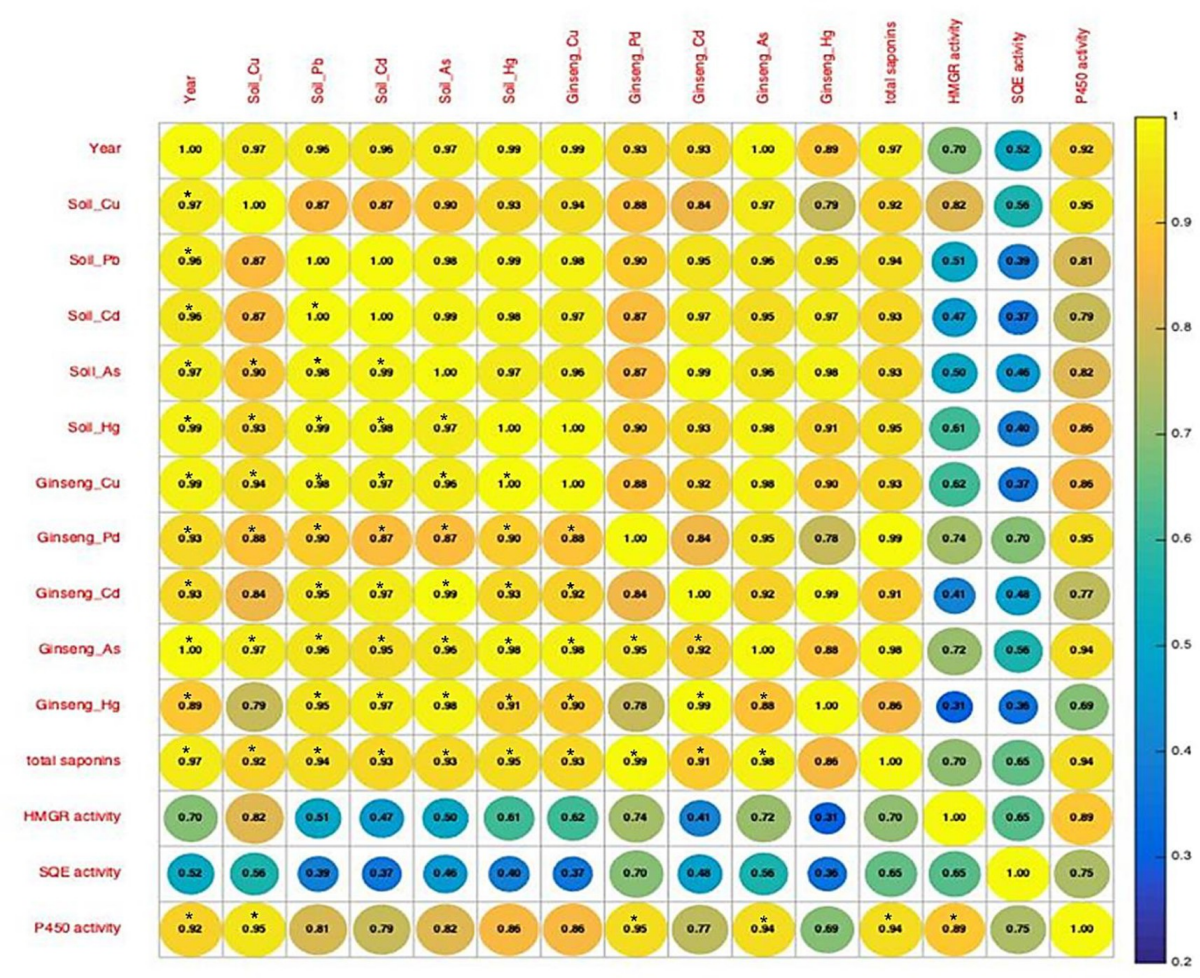
Correlation between ginsenosides content, heavy metal content and enzyme activity in ginseng over time. Colors and Numbers represent the strength of the correlation. * represents P at the 0.05 level.

In the present study, a significant correlation was found between heavy metal elements in ginseng and the growing soil, suggesting that ginseng can absorb heavy metals from the soil (Fig 4). Moreover, we found that heavy metal content in the soil could directly affect the total ginsenosides content in ginseng, as evidenced by a significant correlation between them. Although no significant correlations between heavy metals in the soil and HMGR and SQE activity were found, Cu soil content and total ginsenosides were significantly correlated with CYP450 activity.

**Fig 5:**
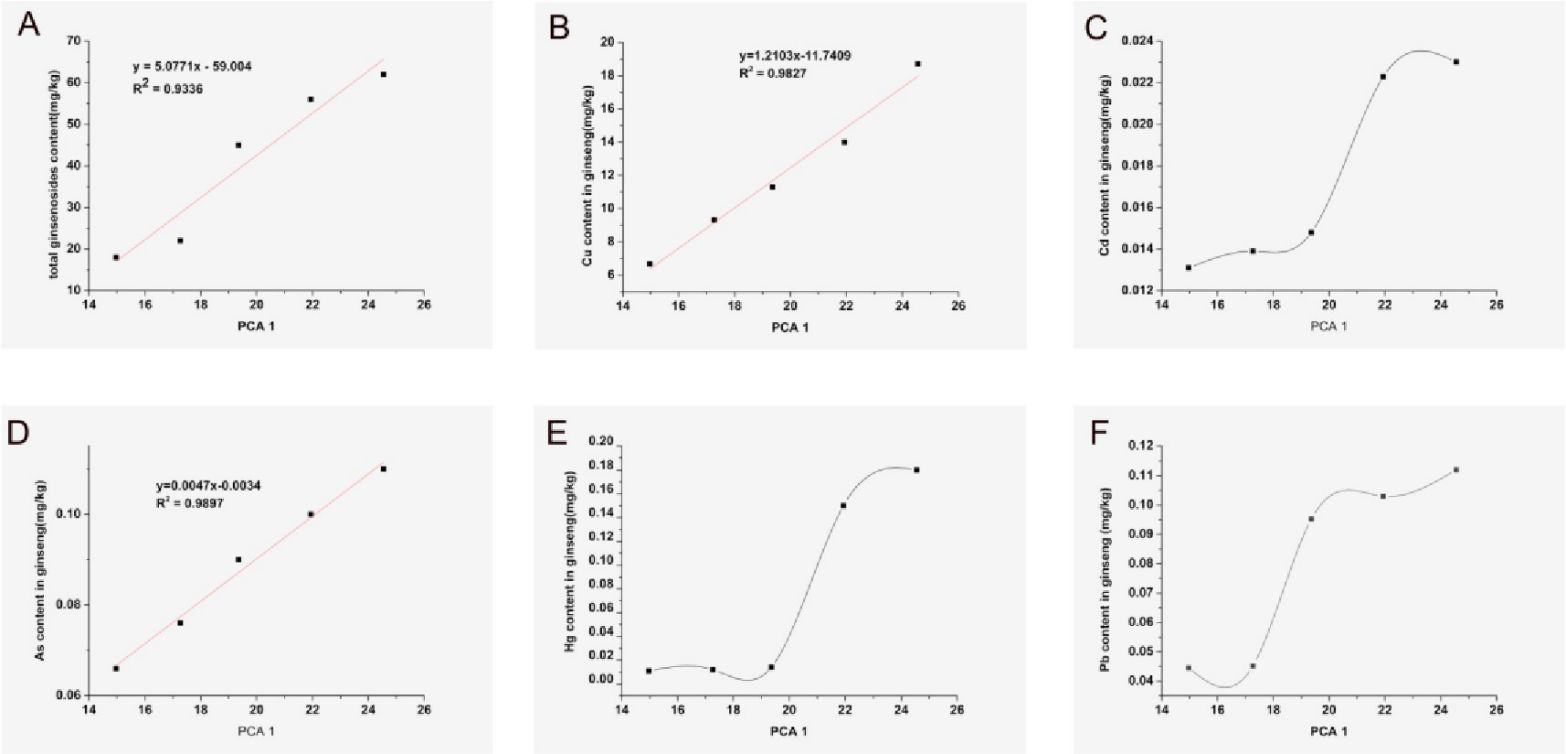
Effect of PCA1 on ginsenoside content and heavy metal content in ginseng. A: The relationship of total ginsenosides content with PCA1. B: The relationship of Cu content in ginseng with PCA1. C: The relationship of Cd content in ginseng with PCA1.D: The relationship of As content in ginseng with PCA1.E: The relationship of Hg content in ginseng with PCA1.F: The relationship of Pb content in ginseng with PCA1.

**Fig 6:**
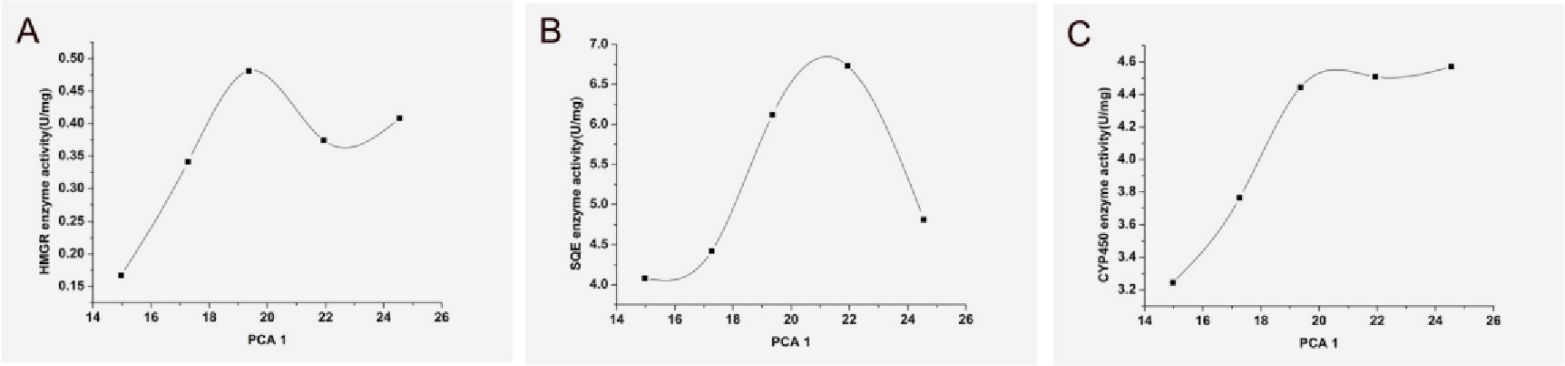
Effect of PCA1 on the activity of rate-limiting enzymes in the synthesis of ginsenosides. A: The relationship of HMGR enzyme activity in ginseng with PCA1;B: The relationship of SQE enzyme activity in ginseng with PCA1;C The relationship of CYP450 enzyme activity in ginseng with PCA1.

It has been established that active dimension reduction by PCA can reduce the number of feature quantities based on data characteristics and analytical needs. At the same time, the "essence" information in the variables can be found. Therefore, we built a PCA model to predict changes in various ginseng elements. We found that PCA1 had a linear relationship with total ginsenosides, Cu, and As in ginseng (Fig 5), with correlation coefficients (r^2^) of 0.9336, 0.9827, and 0.9897, respectively. This linear relationship may enable us to predict the content of ginsenosides by measuring the heavy metal content in ginseng soil. However, the PCA plot indicated a tendency for increasing Cd, Hg and Pb content with ginseng growth, which may be related to the ability of ginseng to absorb nutrients from the soil. Therefore, given that the crucial stage of ginseng growth is from the third to fifth year, its ability to absorb elements in the soil during this period is relatively stronger, even though ginseng does not selectively absorb these, including heavy metals. For the rate-limiting enzymes in the synthesis of ginsenosides (Fig 6), the PCA plot showed an increasing and then decreasing trend for both HMGR and SQE, but CYP450 was the first to increase and stabilize. These phenomena indicated that heavy metals in the soil exerted the greatest influence on enzyme activity in 3 to 5-year-old ginseng plants, which is the rapid growth phase, with high enzymatic activity to synthesize the substances needed for its growth.

## Discussion

An analytical model for the effect of heavy metals on ginseng was established in this study. As a well-known medicine, ginseng represents one of the most valuable medicinal crops globally, well recognized for its "amazing medicinal values" in human health, such as anticancer, heart-protective and neuroprotective properties for thousands of years [25–27]. However, heavy metals are currently a serious problem that restricts this valuable crop’s growth and application. Notwithstanding that there is ample evidence to suggest that heavy metals can influence the growth of plants, few studies have explored the influence of the accumulation of heavy metals in ginseng and soils over time. Herein, we selected 2- to 6-year-old ginseng and their soil for research since 5-6-year-old ginseng can be used for medicinal purposes. Over the years, different detection methods have been established for heavy metals commonly found in ginseng and soil. Meanwhile, ginsenosides and enzyme activity can be detected to evaluate ginseng growth [28].

Heavy metals have different effects on ginsenosides and enzyme activities with different time exposures. In this study, we found a significant correlation between the content of heavy metals in soil and ginseng, which indicated that ginsengs absorb heavy metals in the soil during growth [29]. Three rate-limiting enzyme enzymes are involved in ginsenoside biosynthesis, namely HMGR [30], SQE [31, 32], and CYP450 [24, 33] can affect the production of ginsenosides and play an important catalytic role in the synthesis of ginsenosides [16, 34]. With an increase in ginseng plant ages, increased heavy metal accumulation was observed and the activities of the three enzymes were inhibited to different degrees, especially in the 5 and 6-year-old ginseng. Meanwhile, a relative decrease in the growth rate of ginsenosides was observed during this period, compared to previous years, suggesting that the accumulation of heavy metals can inhibit enzyme activity during the fifth and sixth years. PCA analysis also substantiated that the fastest growing stage of ginseng occurs from the third to fifth year. Peak enzyme activity was observed during this period with a fast heavy metal absorption rate. The discovery of these results is of great significance for ginseng cultivation. Importantly, we found that enzyme activity was influenced by heavy metals, consistent with the literature [35, 36]. Further analysis revealed that Cu in the soil was significantly correlated with the ginsenosides content and the activity of CYP450 in ginseng of different plant ages, suggesting that Cu has a positive effect on ginseng growth [10]. These results were consistent with the important role of copper in CYP450, which could activate the CYP450 enzyme [37, 38]. Moreover, a linear relationship suggested that heavy metal content in soil could predict ginsenosides, Cu and As content in ginseng. Nonetheless, further studies are required to increase the robustness of these findings.

## Conclusion

This research successfully established methods to determine the content of heavy metals in ginseng of different ages and its growing soil and to measure the activity of ginsenosides and their rate-limiting enzymes. We demonstrated that ginsenoside content increased the fastest from the third to the fifth year of growth, and ginseng exhibited high absorption of heavy metals from the soil. The activities of rate-limiting enzymes exhibited different responses to heavy metals in the soils of ginseng of different plant ages. The gradual accumulation of heavy metals in soils eventually inhibited enzyme activity and ginsenoside content in the fifth to sixth year. Moreover, we found that the metallic element Cu could affect the activity of CYP450, exerting a positive effect on ginseng growth. Interestingly, the content of heavy metals in the soil may be a useful predictor for ginsenosides, Cu and As content in ginseng. Findings of the present study emphasize the need to assess changes in heavy metal content in the soil in the first 3–5 years of ginseng growth to ensure the quality and value of ginseng. These findings provide the foothold for future research on ginseng and can assist ginseng growers and industries in producing high-quality ginseng products.

## Supporting information

S1 Fig Standard curves established for the quantification of different heavy metals. (DOCX)
