# Supplementary material for: Ginseng of different ages is affected by the accumulation of heavy metals in ginseng soil
Source: PLoS One. 2022 Jun 13;17(6):e0269238. doi: 10.1371/journal.pone.0269238 (PMC9191705; doi:10.1371/journal.pone.0269238)
Supplement: S1 Fig — (DOCX) [file pone.0269238.s001.docx]

**Supporting information**

**Ginseng of different ages is affected by the accumulation of heavy metals in ginseng soil**

Juxin Yin^1^, Jianjian Zhuang^3*^, Xin Zhang^2^, Chaojian Xu^2^, Shaowu Lv^2^^*^

1 School of information and Electrical Engineering, Zhejiang University City College, Hangzhou 310015，People’s Republic of China

2 Key Laboratory for Molecular Enzymology and Engineering of the Ministry of Education, College of Life Science, Jilin University, Changchun 130000, China

3 Department of Clinical Pharmacology, Key Laboratory of Clinical Cancer Pharmacology and Toxicology Research of Zhejiang Province, Affiliated Hangzhou First People's Hospital, Cancer Center, Zhejiang University School of Medicine, Hangzhou, Zhejiang 310006, China *Correspondence: zhuangjianjian1234@163.com; lvsw@jlu.edu.cn


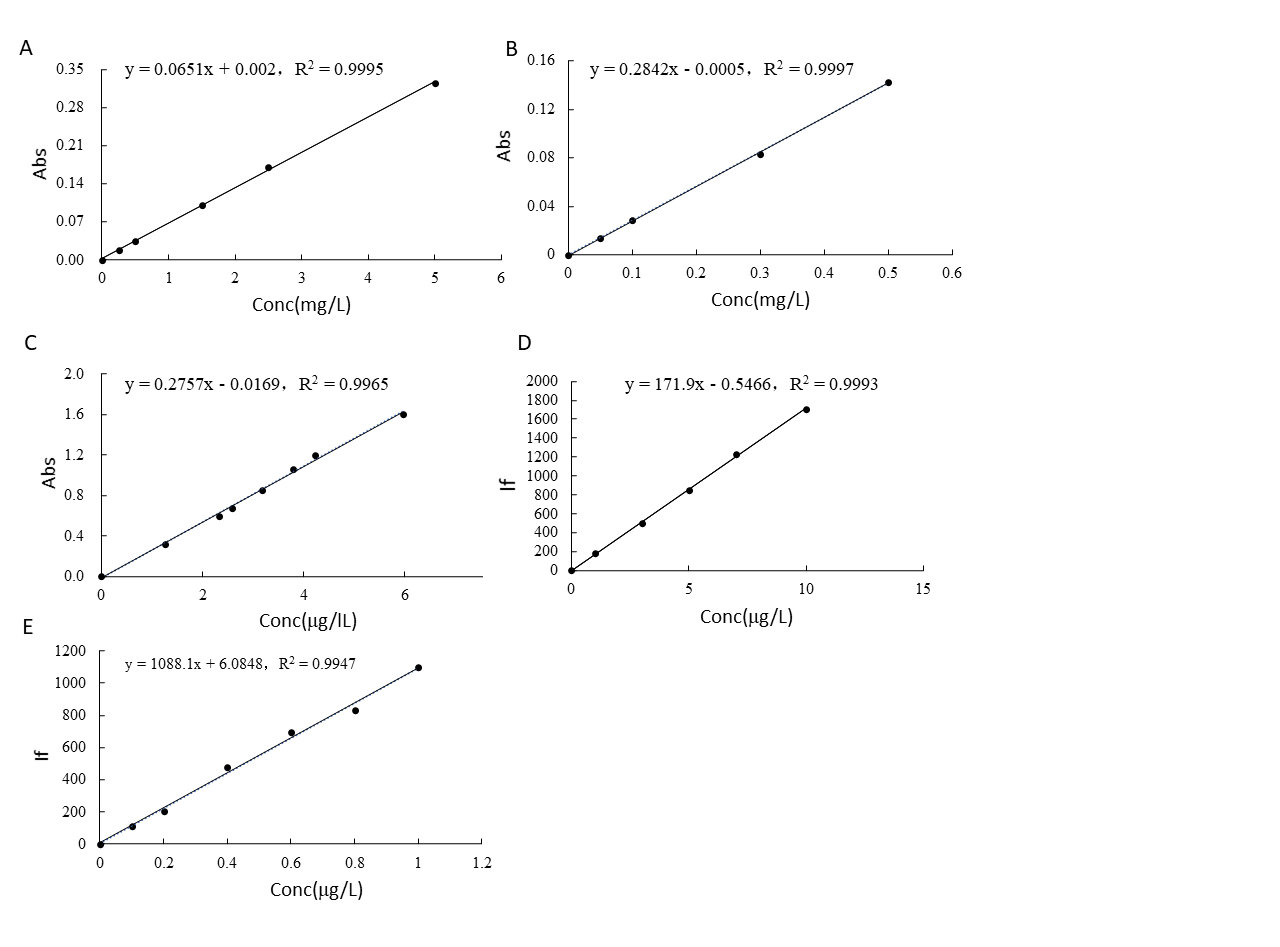


S1 Fig. Standard curves established for the quantification of different heavy metals.

A:Cu, y = 0.0651x + 0.002，R^2^ = 0.9995;

B:Cd, y = 0.2842x - 0.0005，R^2^ = 0.9997;

C:Pb, y = 0.2757x - 0.0169，R^2^ = 0.9965;

D:As, y = 171.9x - 0.5466，R^2^ = 0.9993;

E:Hg, y = 1088.1x + 6.0848，R^2^ = 0.9947.
